# Persistent infection due to a small-colony variant of *Burkholderia pseudomallei* leads to PD-1 upregulation on circulating immune cells and mononuclear infiltration in viscera of experimental BALB/c mice

**DOI:** 10.1371/journal.pntd.0005702

**Published:** 2017-08-18

**Authors:** Jia-Xiang See, Samudi Chandramathi, Mahmood Ameen Abdulla, Jamuna Vadivelu, Esaki M. Shankar

**Affiliations:** 1 Department of Medical Microbiology, Faculty of Medicine, University of Malaya, Lembah Pantai, Kuala Lumpur, Malaysia; 2 Department of Biomedical Sciences, Faculty of Medicine, University of Malaya, Lembah Pantai, Kuala Lumpur, Malaysia; 3 Center of Excellence for Research in AIDS (CERiA), University of Malaya, Lembah Pantai, Kuala Lumpur, Malaysia; 4 Division of Infection Biology, Department of Life Sciences, School of Basic & Applied Sciences, Central University of Tamil Nadu, Thiruvarur, India; Institut Pasteur, FRANCE

## Abstract

**Background:**

Melioidosis is a neglected tropical disease endemic across South East Asia and Northern Australia. The etiological agent, *Burkholderia pseudomallei* (*B*.*pseudomallei*), is a Gram-negative, rod-shaped, motile bacterium residing in the soil and muddy water across endemic regions of the tropical world. The bacterium is known to cause persistent infections by remaining latent within host cells for prolonged duration. Reactivation of the recrudescent disease often occurs in elders whose immunity wanes. Moreover, recurrence rates in melioidosis patients can be up to ~13% despite appropriate antibiotic therapy, suggestive of bacterial persistence and inefficacy of antibiotic regimens. The mechanisms behind bacterial persistence in the host remain unclear, and hence understanding host immunity during persistent *B*. *pseudomallei* infections may help designing potential immunotherapy.

**Methodology/Principal findings:**

A persistent infection was generated using a small-colony variant (SCV) and a wild-type (WT) *B*. *pseudomallei* in BALB/c mice via intranasal administration. Infected mice that survived for >60 days were sacrificed. Lungs, livers, spleens, and peripheral blood mononuclear cells were harvested for experimental investigations. Histopathological changes of organs were observed in the infected mice, suggestive of successful establishment of persistent infections. Moreover, natural killer (NK) cell frequency was increased in SCV- and WT-infected mice. We observed programmed death-1 (PD-1) upregulation on B cells of SCV- and WT-infected mice. Interestingly, PD-1 upregulation was only observed on NK cells and monocytes of SCV-infected mice. In contrast, cytotoxic T-lymphocyte-associated antigen-4 (CTLA-4) downregulation was seen on NK cells of WT-infected mice, and on monocytes of SCV- and WT-infected mice.

**Conclusions/Significance:**

The SCV and the WT of *B*. *pseudomallei* distinctly upregulated PD-1 expression on B cells, NK cells, and monocytes to dampen host immunity, which likely facilitates bacterial persistence. PD-1/PD-L1 pathway appears to play an important role in the persistence of *B*. *pseudomallei* in the host.

## Author summary

*B*. *pseudomallei* is a bacterium that causes melioidosis, a disease endemic in Southeastern Asia and Northern Australia. It is estimated that melioidosis leads to 89,000 deaths worldwide each year. Nevertheless, melioidosis continues to remain a neglected tropical disease that is not even on the list of neglected tropical diseases of the World Health Organization. Furthermore, the disease has a high mortality and recurrence rate, which can be up to 40% and 13%, respectively. It has also been well documented that *B*. *pseudomallei* causes latent/persistent infections for a prolonged period without showing apparent symptoms in the infected individual. The mechanisms that are responsible for bacterial persistence in the host remain unclear. Our results demonstrated that *B*. *pseudomallei* were able to upregulate PD-1 expression on B cells, NK cells, and/or monocytes during persistent diseases, which likely diminish optimal host immunity. The weakened host immunity in turns facilitates persistence of the bacterium. Interestingly, the SCV had a higher PD-1 expression on distinct immune cells compared to the WT, which might explain its frequent association with persistent infections. Immunotherapies by targeting PD-1/PD-L1 pathway could serve as a better treatment than the conventional antibiotic regimens, which cause a high rate of recurrence in melioidosis patients.

## Introduction

*Burkholderia pseudomallei* (*B*. *pseudomallei*) is the causative agent of melioidosis, an infectious disease, endemic across parts of South East Asia and Northern Australia [1]. Despite causing an estimated 89,000 deaths worldwide annually, melioidosis still remains a neglected tropical disease [2]. Being a major cause of community-acquired sepsis, melioidosis has a high mortality rate up to 40% [3]. Common routes of infection include percutaneous inoculation, inhalation, and/or ingestion of contaminated particles or aerosols [4]. Although melioidosis can manifest diverse symptoms such as pneumonia and abscesses in various organs including the brain, bacteremic melioidosis with pneumonia commonly leads to early mortality [5,6]. Apart from acute infection, *B*. *pseudomallei* can cause persistent disease with little or no clinical symptoms over a prolonged period of latency in the host, and only reactivate after years [7–9]. This suggests the likelihood of *B*. *pseudomallei* to reactivate only when the host immunity wanes. Indeed, *B*. *pseudomallei* can be considered also as an opportunistic pathogen, as melioidosis patients are commonly individuals with at least one or more underlying diseases (~80%) and the elderly [3]. Moreover, recurrence rates in patients can be up to ~13% despite appropriate antibiotic treatments[10], suggestive of bacterial persistence and inefficacy of antibiotic regimens. The mechanisms behind bacterial persistence in the host remain unclear.

Small-colony variants (SCVs) representing a sub-population of bacteria have been frequently associated with persistent infections [11–15]. As the name implies, SCVs are slow-growing and form pin-point colonies after 24–72 hours of incubation on agar medium [16]. Although the SCVs of *Staphylococcus aureus* (*S*. *aureus*) remain the most extensively studied variant, the morphotypes have also been described in many other bacteria including *B*. *pseudomallei*. SCVs are known to be relatively more resistant to antibiotics compared with their wild-type (WT) counterparts [17]. In *B*. *pseudomallei*, SCVs were reported to display a greater degree of drug resistance [18]. To the best of our knowledge, we are the only group till date that attempted to study WT and SCVs of *B*. *pseudomallei*. Our proteomic studies revealed that SCVs and WT pre- and post-infection of A549 lung epithelial cells showed distinct expressions of proteins involved in adhesion, invasion, and virulence (Al-Maleki et al., 2014; Al-Maleki et al., 2015). More importantly, our previous study also demonstrated that SCVs and WT triggered distinct host immune responses during persistent *B*. *pseudomallei* infections. Another study also demonstrated that *B*. *pseudomallei* can switch to different morphotypes during stress, and have distinct abilities to persist *in vitro* and *in vivo* [19]. Hence, these pieces of evidence together suggest that SCVs and WT could play different roles in persistent clinical melioidosis.

Programmed death-1 (PD-1) negatively regulates T cell functions, as its engagement with its ligand PD-L1 and PD-L2 arrest T cell proliferation, cytokine secretion, and cytolytic functions [20]. PD-1 is by far the best characterized co-inhibitory molecule associated with T-cell exhaustion in chronic viral infections [21,22]. Apart from chronically-infecting viruses [23–25], many bacteria that cause persistent infections, such as *Mycobacterium tuberculosis* and *Helicobacter pylori (H*. *pylori)*, are known to upregulate PD-1 and PD-L1 [26–30]. Persistent *B*. *pseudomallei* infections in BALB/c mice also led to PD-1 upregulation on CD4+ and CD8+ T cells, suggestive of T cell exhaustion. This is in line with a previous study that reported on PD-L1 upregulation in polymorphonuclear neutrophils infected with *B*. *pseudomallei*, which consequently inhibited CD4+ T-cell functions as well [31]. These results suggest an important role of PD-1/PD-L1 pathway that might potentially be exploited by *B*. *pseudomallei* to facilitate persistence in the host. While the role of PD-1 in functional exhaustion is clearly established in T cells, accumulating lines of evidence indicate that PD-1 negatively regulates the functions of B cells, natural killer (NK) cells, and monocytes [32–37].

Cytotoxic T-lymphocyte-associated antigen-4 (CTLA-4) represents another co-inhibitory molecule that is inducibly expressed on T cells. CTLA-4 is homologous to CD28 (the co-stimulatory molecule that provides second signal for T cell activation), and inhibits T cell activation [38]. Both CTLA-4 and CD28 engage with two cognate ligands, B7-1 (CD80) and B7-2 (CD86), although CTLA-4 binds with a greater affinity [39]. Similar to PD-1, the role of CTLA-4 has been extensively studied in T cells. CTLA-4 upregulation on T cells has been well documented in hepatitis B (HBV) and human immunodeficiency virus (HIV) infections [38,40]. In bacterial infections, CTLA-4 has been reported to cause T cell anergy especially in *H*. *pylori* infections in mice, and pathogen clearance was improved following the blockade of CTLA-4 [41]. Notwithstanding, the role of CTLA-4 is well-studied in T cells, its role in other immune cells rather remains ambiguous. To date, very few studies have demonstrated that CTLA-4 inhibits the functions of B cells, NK cells, and monocytes [42–45]. Therefore, it is conceivable to hypothesize that PD-1 and CTLA-4 could dampen host immune responses leading to establishment of persistent infections. We demonstrated previously that persistent *B*. *pseudomallei* infections can lead to an increased expression of PD-1 on CD4+ and CD8+ T cells [46]. Here, we aimed to investigate into B cell and innate cell responses, including PD-1 and CTLA-4 expressions, during experimental persistent *B*. *pseudomallei* infections. We proposed that persistent *B*. *pseudomallei* infections can lead to upregulation of PD-1 on B cells, NK cells, and monocytes, resulting in suboptimal host immune responses. *B*. *pseudomallei* employs PD-1/PD-L1 pathway as an immune exhaustion strategy to persist in the host.

## Materials and methods

### Ethics statement

All mouse experiments were conducted according to the guidelines of the University of Malaya Animal Care and Use Policy (UM ACUP), and the protocols were reviewed and approved by the Animal Experimental Unit of University of Malaya, Kuala Lumpur, Malaysia (Ref. No.: 2014-08-05/MMB/R/JSV). The Animal Experimental Unit of University of Malaya is accredited by the Association for Assessment and Accreditation of Laboratory Animal Care (AAALAC), and conforms to all government laws and regulations. It provides for approved research and teaching activities, and safeguards the health and welfare of staff and students involved in scholarly activities using animals or animal parts derived from animals. Animals were maintained with controlled temperature, 12h light/dark cycles and given water and feed *ad libitum*. All efforts were made in order to minimize animal suffering. In addition, all bacterial isolates used in this study were analyzed anonymously.

### Bacterial identification

A clinical isolate of *B*. *pseudomallei* from a melioidosis case in University Malaya Medical Center (UMMC) isolated as previously described was used in the study [47]. The isolate, when cultured on agar medium at 37°C, was found to differentiate into two colony morphotypes, OB (WT, INSDC: APLK00000000.1) and OS (SCV, INSDC: APLL00000000.1). OB and OS were characterized using a commercial analytical profile index API 20NE (BioMėrieux) test and an *in house* PCR assay specific for *B*. *pseudomallei* [48]. The two morphotypes were cultured on nutrient agar and a single colony of each morphotype was inoculated into Luria-Bertani (LB) broth (Becton Dickinson, Franklin Lakes, New Jersey, USA) at 37°C overnight in a shaker incubator at 200 revolutions per minute (rpm). Following culture, glycerol (Acros Organics, Geel, Belgium) with a final concentration of 30% (v/v) was added to the LB cultures and stored at -80°C as a stock culture for the entire duration of the study.

### Bacterial inoculum

Bacterial inoculum of OB and OS was prepared as previously described [46]. Briefly, a single colony of OB and OS from nutrient agar was cultured in LB broth and incubated at 37°C overnight at 200rpm. Later, overnight cultures were adjusted to an OD_600_ of 0.05 with LB broth and incubated under similar conditions. Cultures that reached the mid-logarithmic phase (OD_600_ 0.5–0.7) were harvested, washed, and re-suspended in phosphate-buffered saline (PBS). Subsequently, the bacterial suspensions were ten-fold serially diluted with PBS until the desired inoculum was obtained. The inoculum was plated on nutrient agar to enumerate colony-forming units (CFUs).

### Persistent infection

Seven to eight-week-old female BALB/c mice obtained from University Putra Malaysia were used in the experiments. All mice were acclimatized for two weeks prior to infection. Mice were under *ad libitum* feeding conditions. Mice were anaesthetized with isoflurane (Piramal Healthcare Ltd, India), and 10μL of bacterial inoculum was administered via the intranasal route. A persistent *B*. *pseudomallei* infection was generated as described previously with minor modifications [49]. Sub-lethal bacterial dose (~2–8% of LD_50_) was determined as suggested by Goodyear et al. that used ~5% of LD_50_ to generate persistent infections in BALB/c mice. Recently, we confirmed that persistent infections with the sub-lethal bacterial dose led to bacterial colonization in the lungs, livers or spleens for ≥60 days by CFU enumeration of these organs, and development of macroscopic hepatic and splenic abscesses in infected mice [46]. Groups of six mice were infected with OB and OS morphotypes, respectively. Only mice that survived for ≥60 days were sacrificed for use in the downstream experiments. One experiment was performed to collect organ samples (n = 4 per group) from infected (OB or OS) and uninfected mice, respectively for histopathological analysis. Two independent experiments were performed in order to collect adequate sample size (n = 6 per group) from OB-infected, OS-infected, and uninfected mice for analysis of immune cells. Mice inoculated with PBS were used as controls, and will be referred to as uninfected mice for simplicity.

### Peripheral blood mononuclear cells (PBMCs)

Mice with persistent *B*. *pseudomallei* infections were anaesthetized with isoflurane, and blood was drawn via terminal cardiac puncture. Heparinized blood samples were diluted with PBS at a 1:1 ratio. PBMCs were isolated as described [50,51]. Briefly, PBMCs were prepared by density-gradient centrifugation over Ficoll-Paque (Sigma Aldrich). PMBC layer was obtained and washed twice with PBS. Cell viability was determined by 0.4% trypan blue (Life Technologies) staining.

### Haemotoxylin and eosin (H & E) staining

Lungs, livers, and spleens from mice were harvested after withdrawal of blood, and fixed immediately in 10% neutral buffered formalin for 24 hours. Organs were processed as parafilm blocks, followed by the H & E staining, and examined using a microscope. Representative images for each visceral organ were captured.

### Polychromatic flow cytometry

PBMCs (1x10^6^ cells in each tube) were stained with Alexa Fluor 488 hamster anti-mouse CD3e (BD Biosciences, clone 145-2C11), Pe-Cy7 rat anti-mouse CD4 (BD Biosciences, clone GK1.5), APC-H7 rat anti-mouse CD8a (BD Biosciences, clone 53–6.7), APC hamster anti-mouse PD-1 (BD Biosciences, clone J43), and PE hamster anti-mouse CTLA (BD Biosciences, clone UC10-4F10-11), Fixable Viability Stain 510 (BD Biosciences, cloneR35-95). Corresponding isotype control for each antibody was prepared for appropriate setting of gates during multicolor flow cytometry analysis. All antibodies were pre-titrated for optimal working concentration. Data were acquired on an 8-color FACSCanto II immunocytometry system (BD Biosciences) with BD FACSDiva software (BD Bioscience). Data were exported from BD FACSDiva and analyzed using Flowjo software version 10 (Tree Star, Oregon, USA).

### B cell, NK cell, and monocyte immunophenotyping

We used a combination of positive and negative selection strategies to identify B cells, NK cells, and monocytes. NK cells express CD8, and monocytes express low levels of CD4 [52,53]. Therefore, we defined B cells as the lymphocyte population (FSC-A vs SSC-A) that was CD3-, CD4-, and CD8-, NK cells as the lymphocyte population that is CD3- and CD8+, and monocytes as the monocyte population (FSC-A vs SSC-A) that was CD3- and CD4^dim^.

### Statistical analysis

Two-tailed Mann-Whitney U test was used to determine statistical significance among different groups, due to the assumption that samples might not follow Gaussian distribution. Results were illustrated using Box-Whisker Plots. All statistical analyses were done using GraphPad Prism 6 software (La Jolla, California, USA). The level of significance was first set at *P<0.05, ** *P*<0.01, ***P<0.001, and adjusted with appropriate Bonferroni correction.

## Results

### WT *B*. *pseudomallei* exhibited rapid growth on Ashdown’s Agar compared to SCV morphotype

**Fig 1:**
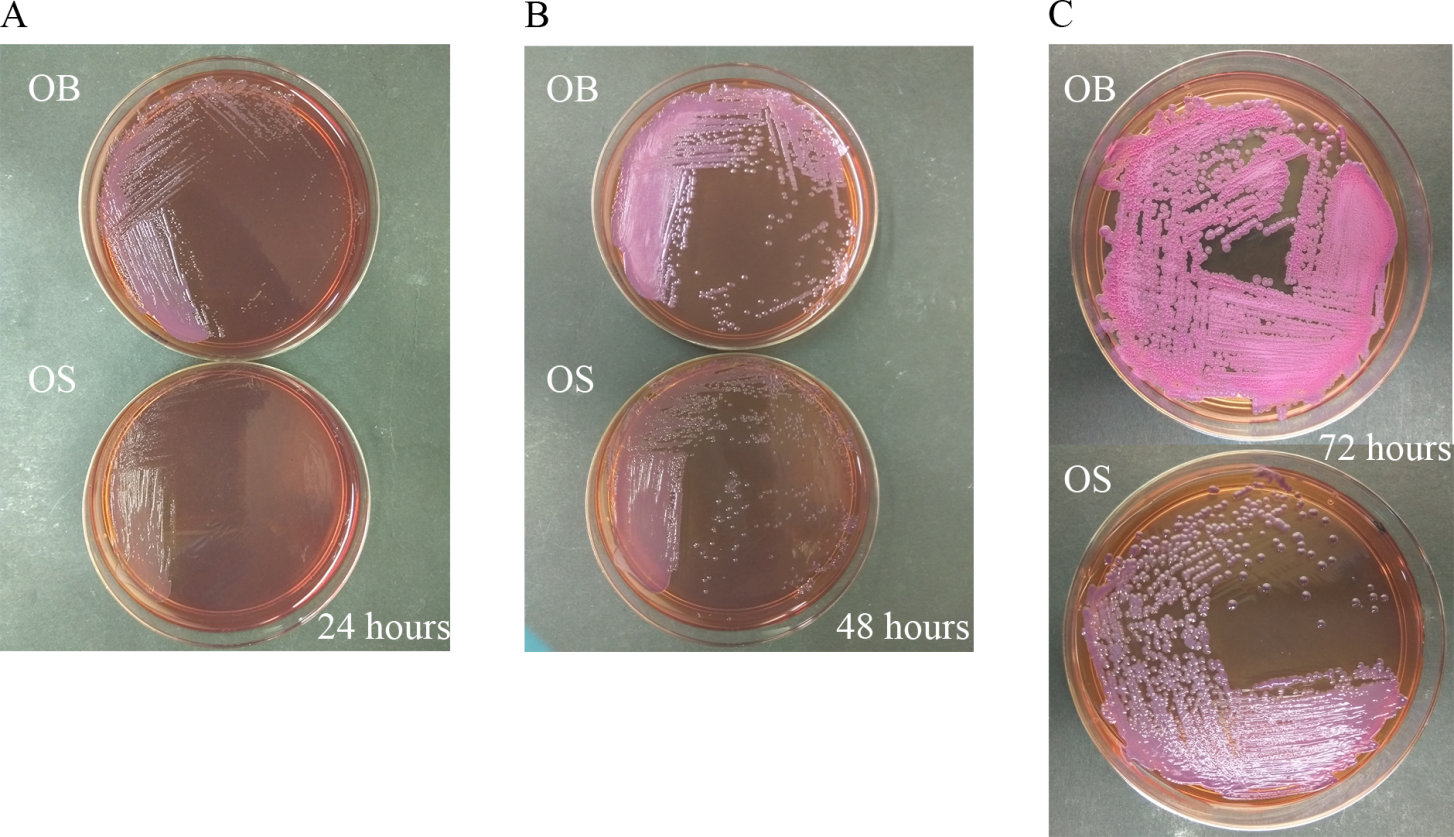
Morphological differences between OB and OS on Ashdown’s agar. **(A & B)** OB colonies were visible after 24 hours of incubation, and continued to grow larger up to 48 hours of incubation. OS morphotype only appeared as small colonies after 48 hours of incubation. **(C)** OB showed rough, wrinkled, and irregular morphology, while OS showed smooth and round morphology after 72 hours of incubation.

Morphological differences between OB and OS morphotypes on Ashdown’s agar, which is a selective agar for *B*. *pseudomallei* [54] were compared following 24 and 48 hours incubation at 37°C under aerobic conditions (Fig 1A & 1B). OB is the WT, whereas OS is the SCV of *B*. *pseudomallei* isolated from the same melioidosis patient. OB developed clear and visible colonies on Ashdown’s agar, while OS could hardly be observed after 24 hours of incubation. Over 48 hours, OB continued to grow larger, whereas OS appeared as small pin-point colonies. This suggests that OS can be differentiated from OB by its slow-growth rate and morphology on Ashdown agar under aerobic conditions. In addition, OB and OS showed distinct morphologies after 72 hours of incubation (Fig 1C). OB appeared as pale purple, rough, wrinkled, and irregular colonies, whereas OS appeared as dark purple, smooth, round and ≥ 2mm diameter colony.

### Persistent *B*. *pseudomallei* infections of BALB/c mice resulted in differential histopathological changes in visceral organs

**Fig 2:**
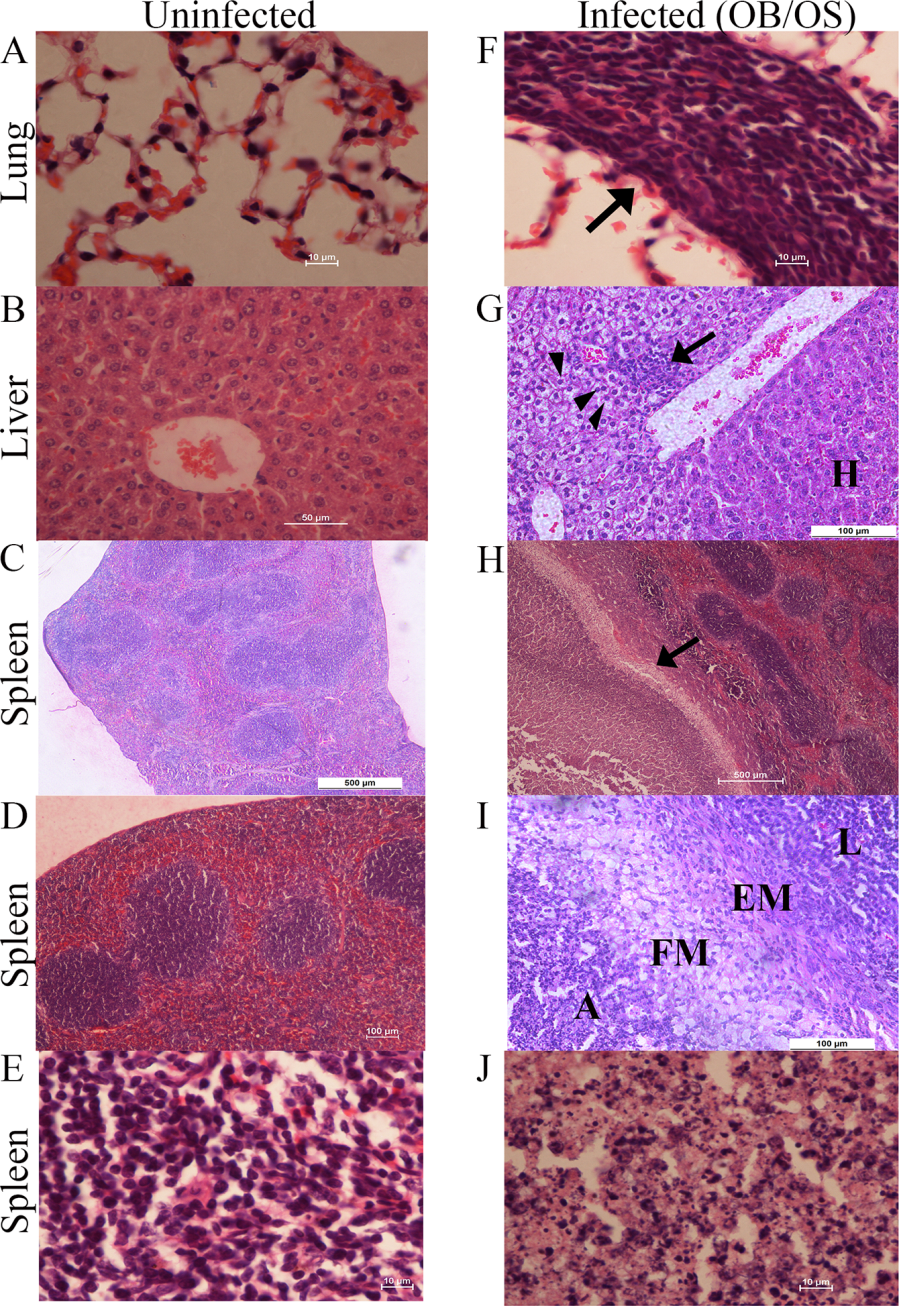
Histopathological changes of lungs, livers, and spleens of mice with persistent *B*. *pseudomallei* infections. **(A)** Lung **(B)** liver and **(C-E)** spleen of uninfected mice show normal histopathology. **(F)** Non-necrotic solid lung lesions characterized by a discrete focus consisting of primarily mononuclear cells (**arrow**). **(G)** Hepatic lesion with predominantly mononuclear cells (**arrow**). Cytoplasmic vacuolation of hepatocytes were observed in the area surrounding the lesion, which is characterized by swelling hepatocytes and clearing cytoplasm (**arrow heads**) Normal hepatocyte-**H**. **(H)** Splenomegaly with large encapsulated abscess cavity (**arrow**). **(I)** The encapsulated abscess-**A** was surrounded by foamy macrophages-**FM**, epithelioid macrophages-**EM**, and lymphocytes-**L**. **(J)** Magnification of the splenic encapsulated abscess, which contains neutrophils, mononuclear cells, bacteria, and necrotic cellular debris. Sections were stained with H & E. Scale bars: 10 μm (A, E, F, J), 50 μm (B & G), 100 μm (D & I) and 500 μm (C & H). Data are representative of one experiment (n = 4 per group).

Lungs, livers, and spleens harvested from OS-infected or OB-infected mice (n = 4 per group) were processed and stained with H & E to investigate the histopathological changes in a persistent *B*. *pseudomallei* infection. Non-necrotic solid lung lesions characterized by a discrete focus consisting primarily of mononuclear cells (Fig 2F) were observed in infected mice.

Livers of infected mice showed lesions with predominantly mononuclear cells (Fig 2G). Cytoplasmic vacuolation was also observed in the hepatocytes of surrounding lesions, characterized by swelling of hepatocytes and clearing of cytoplasm (Fig 2G). Cytoplasmic vacuolation in hepatocytes suggests the likelihood of mild-acute and sub-acute liver injury due to persistent *B*. *pseudomallei* infections.

Several mice showed splenomegaly with large encapsulated abscess cavities containing neutrophils, mononuclear cells, bacteria, and necrotic cellular debris surrounded by a layer of foamy macrophages, followed by epithelioid macrophages and lymphocytes (Fig 2H–2J). On the other hand, some mice (n = 2) after 60 days of persistent *B*. *pseudomallei* infections appeared to have normal red and white pulps with no lesion. Together, these results indicate that intranasal infection of sub-lethal dose *B*. *pseudomallei* causes persistent infections that can lead to histopathological changes and systemic spread of the bacteria from the lungs into the livers and spleens.

### Persistent *B*. *pseudomallei* infections led to increased frequency of NK cells in BALB/c mice

**Fig 3:**
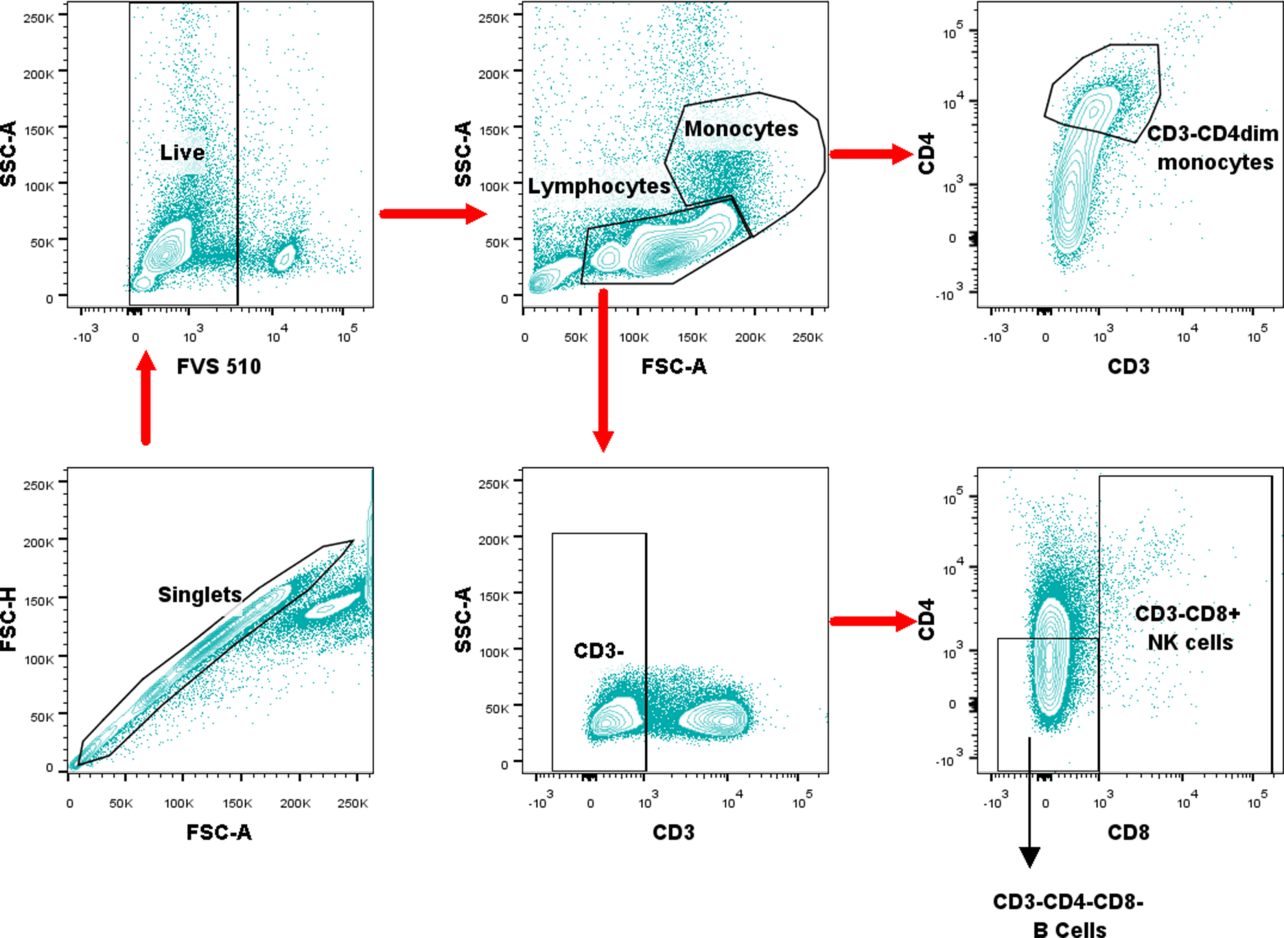
Illustrations of the gating strategy employed in the immunophenotyping of B cells, NK cells, and monocytes. Lymphocytes and monocytes were gated based on forward and side scatter characteristics. B cells were CD3-CD4-CD8-, NK cells were CD3-CD8+, and monocytes were defined as CD3-CD4^dim^. All gates were set using respective isotype controls.

**Fig 4:**
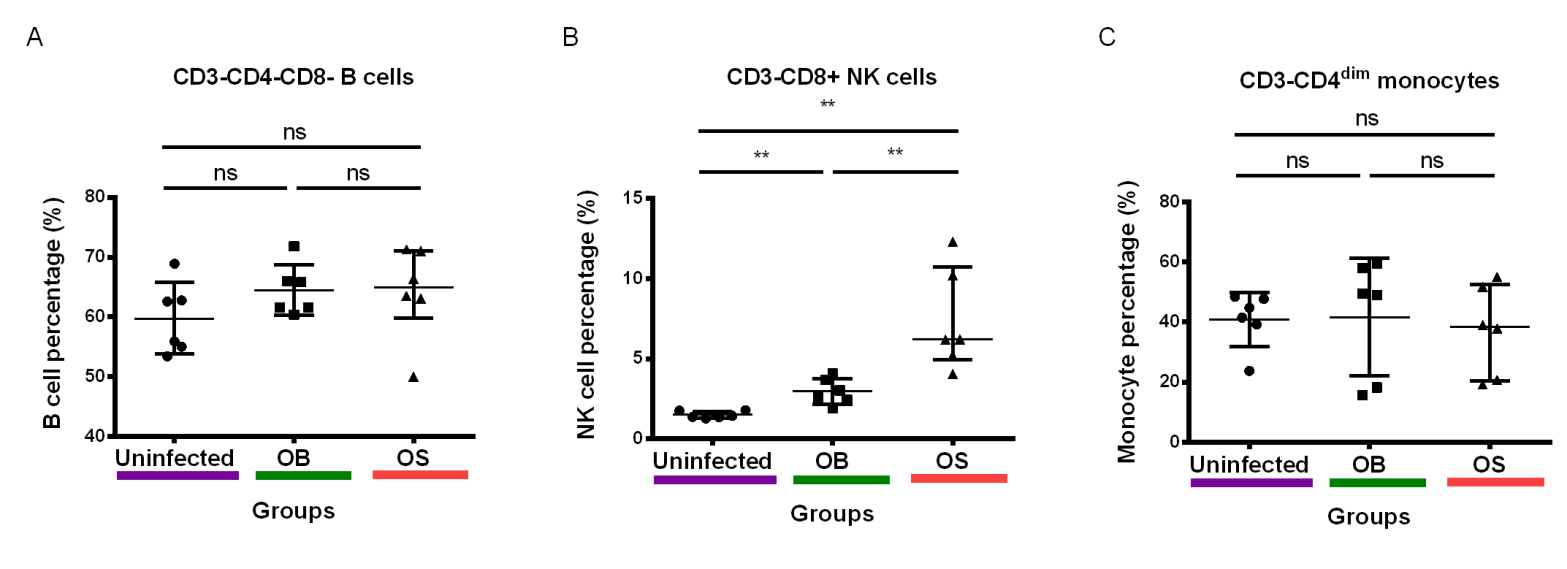
Immune cell frequencies in PBMCs of BALB/c mice infected with *B*. *pseudomallei*. **(A)** B cell **(B)** NK cell and **(C)** monocyte frequencies in PBMCs isolated from uninfected, OB- and OS-infected mice after 60 days of infection. Data representative of two independent experiments (B-D; n = 6 per group). Scatter dot plots show the median value (line), the interquartile range (whiskers). *P* values were calculated using Mann-Whitney U test. *P<0.025, **P<0.005, ***P<0.0005 after Bonferroni correction for 2 comparisons.

Next, we sought to compare the frequencies of B cells, NK cells, and monocytes in the PBMCs of OB-infected, OS-infected, and uninfected mice. Gating strategy for selection of cell population was illustrated (Fig 3). Our results revealed that both OS-infected and OB-infected mice had a higher NK cell frequency relative to uninfected mice (Fig 4B). Interestingly, OS-infected mice had a higher NK cell frequency relative to the uninfected mice. No significant differences were found in monocyte and B cell frequencies among OS-infected, OB-infected, and uninfected mice (Fig 4A & 4C). Together, our results suggest a potential role of NK cell in persistent *B*. *pseudomallei* infections.

### Persistent *B*. *pseudomallei* infections led to upregulation of PD-1, but not CTLA-4, on B cells in BALB/c mice

**Fig 5:**
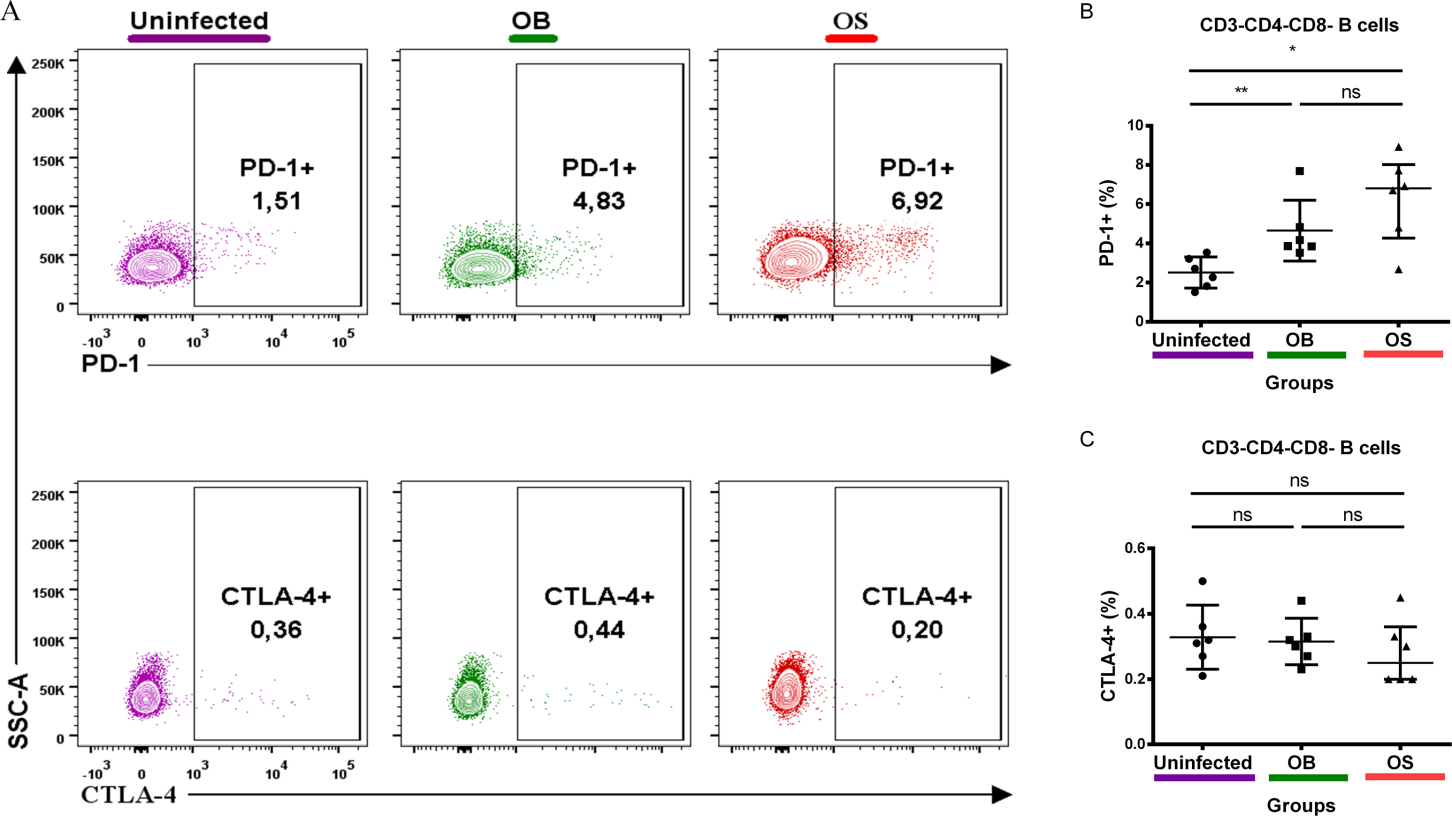
PD-1 and CTLA-4 expressions on Singlet/FVS 510-/Lymph/CD3-/CD4-/CD8- B cells. Representative contour plot of **(A)** PD-1 and CTLA-4 expressions on B cells of uninfected, OB-infected and OS-infected mice after 60 days of infection. **(B)** PD-1+ and **(C)** CTLA-4+ percentage of uninfected, OB-infected and OS-infected mice. Data are pooled from two independent experiments (A-C; n = 6 per group). Scatter dot plots show the median value (line), the interquartile range (whiskers). *P* values calculated using Mann-Whitney U test. *P<0.025, **P<0.005, ***P<0.0005 after Bonferroni correction for 2 comparisons.

PD-1 and CTLA-4 belong to the B7-CD28 superfamily, and their expressions inhibit B cell functions [32,35,39,42,43]. Adaptive immune responses play a paramount role against persistent infections. However, the expression levels of PD-1 and CTLA-4 on B cells have seldom been investigated in *B*. *pseudomallei* infections. We found that the percentage of B cells that expressed PD-1 was increased in OS-infected and OB-infected compared with uninfected mice (Fig 5A & 5B). Nevertheless, no significant changes were found on B cells that expressed CTLA-4 among the three groups studied (Fig 5A & 5C). Taken together, our results indicate that expression of PD-1, but not CTLA-4, could attenuate optimal B cell functions during persistent *B*. *pseudomallei* infections.

### SCV *B*. *pseudomallei* infections of BALB/c mice resulted in the upregulation of PD-1 on NK cells of BALB/c mice

**Fig 6:**
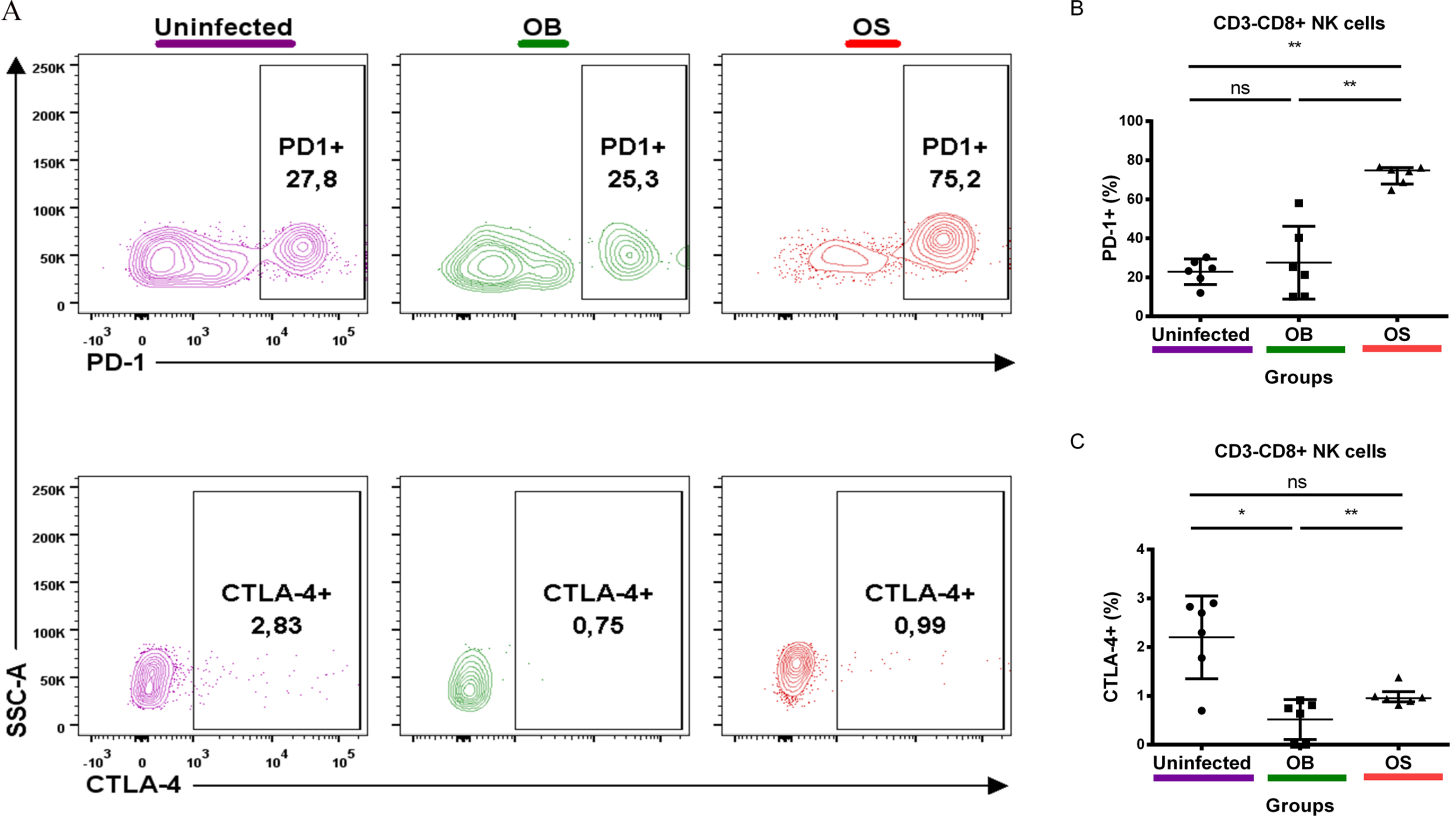
PD-1 and CTLA-4 expressions on Singlet/FVS 510-/Lymph/CD3-/CD8+ NK cells. Representative contour plot of **(A)** PD-1 and CTLA-4 expressions on NK cells of uninfected, OB-infected and OS-infected mice after 60 days of infection. **(B)** PD-1+ and C) CTLA-4+ percentage of uninfected, OB-infected and OS-infected mice. Data are pooled from two independent experiments (**A-C**; n = 6 per group). Scatter dot plots show the median value (**line**), the interquartile range (**whiskers**). *P* values calculated using Mann-Whitney U test. *P<0.025, **P<0.005, ***P<0.0005 after Bonferroni correction for 2 comparisons.

Next, we looked into the innate immunity. PD-1 and CTLA-4 expressions arrest IFN-γ secretion capability of NK cells [34,45]. Thus, we examined the profile of PD-1 and CTLA-4 expressions on NK cells. Interestingly, OS-infected mice had a remarkable increase of NK cells expressing PD-1 relative to WT-infected and uninfected mice (Fig 6A & 6B). However, no changes in NK cells expressing PD-1 were observed between the OB-infected and uninfected mice. Interestingly, OB-infected mice had a lower NK cell frequency that expressed CTLA-4 as compared with OS-infected and uninfected mice (Fig 6A & 6C). Our findings demonstrate that SCV *B*. *pseudomallei* upregulates PD-1 expression on NK cells in mice during persistent infections, suggestive of NK cell exhaustion.

### SCV *B*. *pseudomallei* infection of BALB/c mice culminated in PD-1 upregulation and CTLA-4 downregulation on monocytes

**Fig 7:**
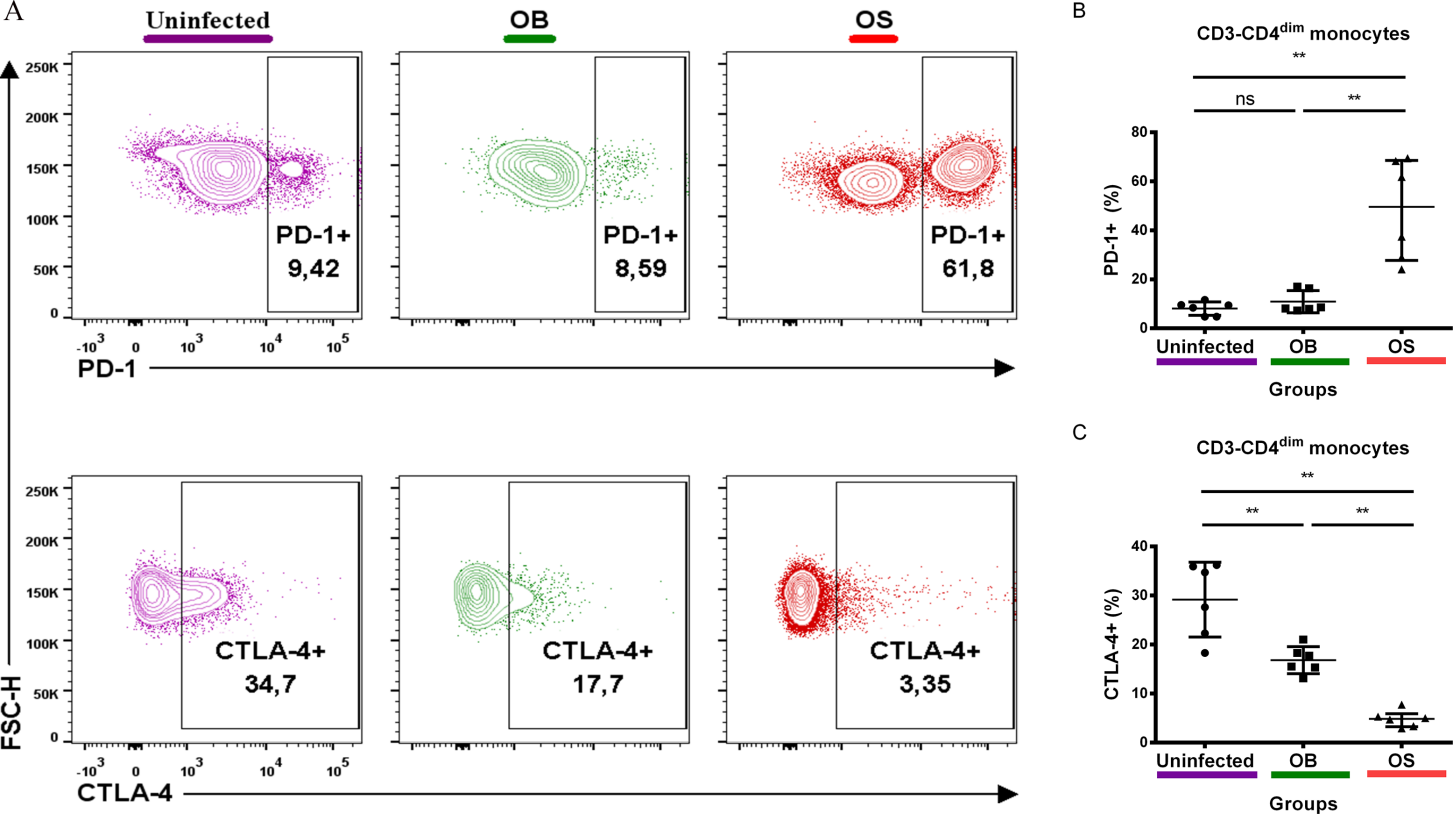
PD-1 and CTLA-4 expressions on Singlet/FVS 510-/Monocyte/CD3-/CD4^dim^ monocytes. Representative contour plot of **(A)** PD-1 and CTLA-4 expressions on monocytes of uninfected, OB-infected and OS-infected mice after 60 days of infection. **(B)** PD-1+ and **(C)** CTLA-4+ percentage of uninfected, OB-infected and OS-infected mice. Data are pooled from two independent experiments (**A-C**; n = 6 per group). Scatter dot plots show the median value (**line**), the interquartile range (**whiskers**). *P* values calculated using Mann-Whitney U test. *P<0.025, **P<0.005, ***P<0.0005 after Bonferroni correction for 2 comparisons.

Finally, we investigated the expressions of PD-1 and CTLA-4 on monocytes, as these two co-inhibitory molecules both negatively regulate monocyte functions [36,37,44]. We observed that OS-infected mice had a higher frequency of monocytes expressing PD-1 compared with OB-infected and uninfected mice (Fig 7A & 7B). No significant differences were observed between OB-infected and uninfected mice. Interestingly, we noticed a remarkable decrease in monocytes expressing CTLA-4 in OS-infected and OB-infected relative to uninfected mice (Fig 7A & 7C). OS-infected mice had a lower frequency of monocytes expressing CTLA-4 compared with OB-infected mice. Together, our results suggest that PD-1 and CTLA-4 are implicated in monocyte functions during persistent *B*. *pseudomallei* infections.

## Discussion

Our OS morphotype was stable and reproducible throughout the experiment, as it did not revert back to WT morphology when cultured from glycerol stock, during growth kinetic study and preparation of inoculum for infection. Our previous study showed that OS grew slower on nutrient agar compared to OB [46]. Moreover, our growth kinetic study demonstrated that OS had a defect in growth *in vitro*, as it grew much slower in Lunia-Bertani (LB) broth and reached a much lower OD_600_ density compared to OB. In this study, OS also grew slower on Ashdown’s agar and had different morphology when compared to OB. We categorized OB as type I (pale purple, irregular and rough colonies) and OS as type III (dark purple and smooth colonies) morphotype according to Chantratita et al. [19].

Despite previous studies on SCV and WT *B*. *pseudomallei* using both *in vitro* and *in vivo* model, literature on the pathogenesis of persistent infections due to SCVs and WT still remains scarce [46,55,56]. Tuchsherr et al. [57] revealed that intracellular infection of endothelial cells with SCVs of *S*. *aureus* did not cause any dramatic change in the genes that regulate innate immune responses compared to WT morphotypes. Nevertheless, the study by Tuchsherr et al. could only explain an acute-like intracellular infection *in vitro* as the infection assay was only conducted for a few hours. Hence, our experimental mouse model serves the purpose of comparing the pathogenesis between SCVs and WT during persistent *B*. *pseudomallei* infections. We previously showed that a higher bacterial burden was observed in spleens, but not in the lungs and livers of mice infected with the SCV compared with the WT morphotype [46]. Besides, mice that survived an infection with the SCV of *B*. *pseudomallei* for two months were more likely to develop macroscopic liver or splenic abscesses compared with the WT morphotype. Significant changes in lungs, despite using the intranasal route for infection were not observed. This observation can be supported by a previous study on the “persistence model” that demonstrated higher bacterial recovery percentage from livers and spleens compared with lungs after intranasal challenge [49].

To date, only Conejero et al. [58] attempted to characterize the histopathological changes in lungs, livers, and spleens in a chronic *B*. *pseudomallei* infection using C57BL/6 mice. In their study, four different types of lung lesions were observed, with two types of them forming granulomas. Additionally, the study also observed pyogranuloma with a necrotic center containing neutrophils surrounded by macrophages, plasma cells, and lymphocytes in the liver. Small pyogranulomas containing neutrophils and macrophages were also common in the liver. Moreover, multifocal to coalescent pyogranulomatous splenitis containing a necrotic center and nonnecrotic microgranulomas consisting epithelioid macrophages were also observed.

In our study, contrary to Conejero et al.’s findings, only one type of lung lesion was observed, which was characterized by a discrete focus of lymphocyte infiltration, with no granuloma (Fig 2F). However, similar findings were observed in many of the lungs of infected mice that had few to no significant lesions. In contrast to Conejero et al., minor hepatic lesions characterized by infiltration of predominantly mononuclear cells in most of the infected mice were observed. Notably, cytoplasmic vacuolation in hepatocytes of several infected mice was observed, suggesting mild-acute and subacute liver injury due to persistent *B*. *pseudomallei* infections (Fig 2G). In contrast to Conejero et al., normal spleen histology for several infected mice was observed in the present study. Several mice with macroscopic abscesses showed splenomegaly and necrotic pyogranulomas containing neutrophils, which was surrounded by a layer of foamy macrophages, followed by epithelioid macrophages and lymphocytes (Fig 2H and 2I).

Different observations compared with Conejero et al. were possibly caused by different strains of *B*. *pseudomallei* and mice used in the study. More importantly, Conejero et al. sacrificed mice with chronic *B*. *pseudomallei* infections after 20 to 60 days of infections, while our study sacrificed mice only after 60 days of persistent infections. The inconsistent duration of sacrificing mice for histopathological investigation in their study might have contributed significantly to different observations. Mice that were sacrificed on the 20^th^ day could have not survived for a longer period due to a more serious *B*. *pseudomallei* infection. This would have led to a more severe histopathological changes and a biased observation. The present study sacrificed mice only after 60 days, which is the period considered as chronic melioidosis [59]. This duration leads to a more accurate and consistent histopathological investigation for persistent *B*. *pseudomallei* infections.

We speculate that the SCV is more likely to cause severe persistent disease, which was reflected by the higher bacterial load in spleens and more abscess formation in livers and spleens compared with the WT [46]. It is illogical that SCVs result in a more severe pathology than WT, as this process will not benefit the bacteria to persist for a longer duration due to massive host immune responses. However, a recent study by Dietrich et al. [60] demonstrated that non-replicating *M*. *tuberculosis* caused a higher CFU and an increased number of granulomas in mouse lungs compared with WT after six weeks. Dietrich et al. suggest that non-replicating *M*. *tuberculosis* might undermine host immunity leading to higher bacterial replication and severe pathology. This might be part of the resuscitation process for non-replicating or dormant bacteria to eventually facilitate its transmission. SCVs are similar to dormant bacteria in certain ways, including resistance to antibiotics and slow/zero growth rate. Our results demonstrate that SCVs might employ the same strategy as dormant *M*. *tuberculosis* by causing a greater degree of pathology in order to facilitate its transmission in persistent *B*. *pseudomallei* infections. This explanation is reasonable as one would anticipate SCVs to relapse in some time in future to transmit the disease.

There are only limited studies on the role of B cells, NK cells, and monocytes in melioidosis. Antibodies against *B*. *pseudomallei* appears to play a less significant role against melioidosis despite that many individuals still show high seropositivity across endemic regions [61]. B cells were found to play a lesser role in protecting against *B*. *pseudomallei* as evident from experiments conducted on B cell-deficient (μMT) mice. Nevertheless, it is now clear that μMT mice still produce B cells that could produce other isotypes, raising doubt of using this model to investigate the role of B cells [62–66]. Moreover, immunized mice which produced a high IgG level after lethal challenge had a survival rate of >80% after 40 days [67], suggestive of a protective role of B cells in experimental *B*. *pseudomallei* infections. Here, we showed an increase in B cells expressing PD-1 in the SCV-infected and WT-infected, compared with uninfected mice. This suggests that *B*. *pseudomallei* could upregulate PD-1 on B cells to limit optimal B cell functions, which likely affect antibody production, and their interaction with follicular Th cells (Tfh) however, may require more investigations.

NK cells are a unique population, as many studies have demonstrated that NK cells capture hallmarks of adaptive immunity including antigen specificity and memory responses [68,69]. In addition, NK cells can be functionally exhausted similar to T cells during chronic diseases [70,71]. NK cells have been identified as the major producer of interferon-γ (IFN-γ) in experimental and human melioidosis [66,72]. In experimental melioidosis, NK cell-derived IFN-γ showed functional redundancy with IFN-γ released by other immune cells in the first two days of infection [66]. Nevertheless, NK cells could still be playing an essential protective role over prolonged periods of *B*. *pseudomallei* infections [66]. Accordingly, our results demonstrated that persistent *B*. *pseudomallei* infections can lead to an increase in NK cell frequency regardless of bacterial morphotype differences. In this study, SCV-infected mice had a higher NK cell frequency as compared with WT-infected mice. Interestingly, only SCV-infected mice showed a higher percentage of PD-1+ NK cells, suggesting NK cell exhaustion. WT-infected mice had a lower frequency of CTLA-4+ NK cells relative to SCV-infected and uninfected mice. However, the role of differential expressions of PD-1 and CTLA-4 on the regulation of NK cell activities warrants further investigation.

Several findings have indicated that monocytes could possibly play a role in *B*. *pseudomallei* infections [73–75]. A recent study on human primary monocytes demonstrated that *B*. *pseudomallei* infections stimulated IL-23 production in these cells [75]. Interestingly, in our earlier study, it was demonstrated that persistent infections with SCV *B*. *pseudomallei* led to an increase in plasma IL-17A. Briefly, IL-23 is essential for inducing the production of IL-17,as well as expanding and stabilizing Th17 cells [76]. Thus, activated monocytes could be the major source of IL-23 in maintaining Th17 cells during persistent *B*. *pseudomallei* infections. Moreover, high mRNA expression of inflammatory genes in monocytes positively correlated with mortality in patients with sepsis due to *B*. *pseudomallei* [74]. Our findings affirmed that SCV-infected mice had a higher PD-1 expression on monocytes compared with WT-infected and uninfected mice. Strikingly, SCV-infected and WT-infected mice had a lower frequency of monocytes expressing CTLA-4 relative to uninfected mice, with the SCV resulting in a lower CTLA-4 expression on monocytes compared with the WT. The observed changes in PD-1 and CTLA-4 expression on monocyte functions would be an interesting future consideration.

In this study, we were not able to use definitive approaches including CD19, CD56, CD14/CD16 to identify B cells, NK cells, and monocytes. However, the markers used in the current study were based on previous studies conducted on HIV infection [52,53]. In addition, we were only able to characterize the expression of PD-1 and CTLA-4 levels on B cells, NK cells, and monocytes without dissecting the functional role of these molecules. Nevertheless, we demonstrated for the first time that persistent *B*. *pseudomallei* infections with SCVs can concurrently lead to PD-1 expression on B cells, NK cells, and monocytes in mice, clearly suggesting host immune exhaustion. Remarkably, SCVs caused a higher PD-1 upregulation on NK cells and monocytes compared with WT. Together with our previous work, we could conclude that SCVs caused PD-1 upregulation on adaptive (T and B cells) and innate immune cells (NK cells or monocytes), while WT caused PD-1 upregulation only on adaptive immune cells. These observations might be due to the more efficient ability of SCVs in causing host immune exhaustion, or in causing a greater pathology as compared with WT. We speculate that SCVs initiate a higher expression of PD-1 to suppress the host immune responses and facilitate their persistence, causing increased bacterial burden. Interestingly, SCVs and WT were shown to cause CTLA-4 downregulation on NK cells and monocytes. It is unclear whether PD-1 upregulation and/or CTLA-4 downregulation are playing the dominant role over the functions of these immune cells. Future studies should be aimed to investigate the functional role of PD-1 and CTLA-4 on various immune cells in *B*. *pseudomallei* infections using murine knockout models and checkpoint inhibitors using *in vivo* experiments.

## References

[1] Cheng AC, Currie BJ (2005) Melioidosis: epidemiology, pathophysiology, and management. Clin Microbiol Rev 18: 383–416. doi: 10.1128/CMR.18.2.383-416.2005 15831829 10.1128/CMR.18.2.383-416.2005 PMC1082802

[2] Limmathurotsakul D, Golding N, Dance DAB, Messina JP, Pigott DM, et al (2016) Predicted global distribution of Burkholderia pseudomallei and burden of melioidosis. Nature Microbiology 1: 15008. 10.1038/nmicrobiol.2015.8 27571754

[3] Wiersinga WJ, Currie BJ, Peacock SJ (2012) Melioidosis. N Engl J Med 367: 1035–1044. doi: 10.1056/NEJMra1204699 22970946 10.1056/NEJMra1204699

[4] Limmathurotsakul D, Peacock SJ (2011) Melioidosis: a clinical overview. Br Med Bull 99: 125–139. doi: 10.1093/bmb/ldr007 21558159 10.1093/bmb/ldr007

[5] Padiglione A, Ferris N, Fuller A, Spelman D (1998) Brain abscesses caused by Burkholderia pseudomallei. J Infect 36: 335–337. 9661950 10.1016/s0163-4453(98)94639-4

[6] Mukhopadhyay A, Lee KH, Tambyah PA (2004) Bacteraemic melioidosis pneumonia: impact on outcome, clinical and radiological features. Journal of Infection 48: 334–338. doi: 10.1016/j.jinf.2003.10.005 15066335 10.1016/j.jinf.2003.10.005

[7] Koponen MA, Zlock D, Palmer DL, Merlin TL (1991) Melioidosis. Forgotten, but not gone! Arch Intern Med 151: 605–608. 2001144 10.1001/archinte.151.3.605

[8] Mays EE, Ricketts EA (1975) Melioidosis: recrudescence associated with bronchogenic carcinoma twenty-six years following initial geographic exposure. Chest 68: 261–263. 1149556 10.1378/chest.68.2.261

[9] Ngauy V, Lemeshev Y, Sadkowski L, Crawford G (2005) Cutaneous melioidosis in a man who was taken as a prisoner of war by the Japanese during World War II. J Clin Microbiol 43: 970–972. doi: 10.1128/JCM.43.2.970-972.2005 15695721 10.1128/JCM.43.2.970-972.2005 PMC548040

[10] Currie BJ, Fisher DA, Anstey NM, Jacups SP (2000) Melioidosis: acute and chronic disease, relapse and re-activation. Trans R Soc Trop Med Hyg 94: 301–304. 10975006 10.1016/s0035-9203(00)90333-x

[11] Kahl B, Herrmann M, Everding AS, Koch HG, Becker K, et al (1998) Persistent infection with small colony variant strains of Staphylococcus aureus in patients with cystic fibrosis. J Infect Dis 177: 1023–1029. 9534977 10.1086/515238

[12] Proctor RA, van Langevelde P, Kristjansson M, Maslow JN, Arbeit RD (1995) Persistent and relapsing infections associated with small-colony variants of Staphylococcus aureus. Clin Infect Dis 20: 95–102. 7727677 10.1093/clinids/20.1.95

[13] von Eiff C, Bettin D, Proctor RA, Rolauffs B, Lindner N, et al (1997) Recovery of small colony variants of Staphylococcus aureus following gentamicin bead placement for osteomyelitis. Clin Infect Dis 25: 1250–1251. 9402396 10.1086/516962

[14] Spanu T, Romano L, D'Inzeo T, Masucci L, Albanese A, et al (2005) Recurrent ventriculoperitoneal shunt infection caused by small-colony variants of Staphylococcus aureus. Clin Infect Dis 41: e48–52. doi: 10.1086/432577 16080075 10.1086/432577

[15] Abele-Horn M, Schupfner B, Emmerling P, Waldner H, Goring H (2000) Persistent wound infection after herniotomy associated with small-colony variants of Staphylococcus aureus. Infection 28: 53–54. 10697795 10.1007/s150100050014

[16] Proctor RA, von Eiff C, Kahl BC, Becker K, McNamara P, et al (2006) Small colony variants: a pathogenic form of bacteria that facilitates persistent and recurrent infections. Nat Rev Microbiol 4: 295–305. doi: 10.1038/nrmicro1384 16541137 10.1038/nrmicro1384

[17] Proctor RA, von Humboldt A (1998) Bacterial energetics and antimicrobial resistance. Drug Resist Updat 1: 227–235. 16904405 10.1016/s1368-7646(98)80003-4

[18] Haussler S, Rohde M, Steinmetz I (1999) Highly resistant Burkholderia pseudomallei small colony variants isolated in vitro and in experimental melioidosis. Med Microbiol Immunol 188: 91–97. 10753061 10.1007/s004300050110

[19] Chantratita N, Wuthiekanun V, Boonbumrung K, Tiyawisutsri R, Vesaratchavest M, et al (2007) Biological relevance of colony morphology and phenotypic switching by Burkholderia pseudomallei. J Bacteriol 189: 807–817. doi: 10.1128/JB.01258-06 17114252 10.1128/JB.01258-06 PMC1797308

[20] Riley JL (2009) PD-1 signaling in primary T cells. Immunol Rev 229: 114–125. doi: 10.1111/j.1600-065X.2009.00767.x 19426218 10.1111/j.1600-065X.2009.00767.x PMC3424066

[21] Keir ME, Butte MJ, Freeman GJ, Sharpe AH (2008) PD-1 and its ligands in tolerance and immunity. Annu Rev Immunol 26: 677–704. doi: 10.1146/annurev.immunol.26.021607.090331 18173375 10.1146/annurev.immunol.26.021607.090331 PMC10637733

[22] Yi JS, Cox MA, Zajac AJ (2010) T-cell exhaustion: characteristics, causes and conversion. Immunology 129: 474–481. doi: 10.1111/j.1365-2567.2010.03255.x 20201977 10.1111/j.1365-2567.2010.03255.x PMC2842494

[23] Wherry EJ, Ha SJ, Kaech SM, Haining WN, Sarkar S, et al (2007) Molecular signature of CD8+ T cell exhaustion during chronic viral infection. Immunity 27: 670–684. doi: 10.1016/j.immuni.2007.09.006 17950003 10.1016/j.immuni.2007.09.006

[24] Barathan M, Gopal K, Mohamed R, Ellegard R, Saeidi A, et al (2015) Chronic hepatitis C virus infection triggers spontaneous differential expression of biosignatures associated with T cell exhaustion and apoptosis signaling in peripheral blood mononucleocytes. Apoptosis 20: 466–480. doi: 10.1007/s10495-014-1084-y 25577277 10.1007/s10495-014-1084-y

[25] Velu V, Shetty RD, Larsson M, Shankar EM (2015) Role of PD-1 co-inhibitory pathway in HIV infection and potential therapeutic options. Retrovirology 12: 14 doi: 10.1186/s12977-015-0144-x 25756928 10.1186/s12977-015-0144-x PMC4340294

[26] Jurado JO, Alvarez IB, Pasquinelli V, Martinez GJ, Quiroga MF, et al (2008) Programmed death (PD)-1:PD-ligand 1/PD-ligand 2 pathway inhibits T cell effector functions during human tuberculosis. J Immunol 181: 116–125. 18566376 10.4049/jimmunol.181.1.116

[27] Singh A, Mohan A, Dey AB, Mitra DK (2013) Inhibiting the programmed death 1 pathway rescues Mycobacterium tuberculosis-specific interferon gamma-producing T cells from apoptosis in patients with pulmonary tuberculosis. J Infect Dis 208: 603–615. doi: 10.1093/infdis/jit206 23661793 10.1093/infdis/jit206

[28] Wu YY, Lin CW, Cheng KS, Lin C, Wang YM, et al (2010) Increased programmed death-ligand-1 expression in human gastric epithelial cells in Helicobacter pylori infection. Clin Exp Immunol 161: 551–559. doi: 10.1111/j.1365-2249.2010.04217.x 20646001 10.1111/j.1365-2249.2010.04217.x PMC2962974

[29] Beswick EJ, Pinchuk IV, Das S, Powell DW, Reyes VE (2007) Expression of the programmed death ligand 1, B7-H1, on gastric epithelial cells after Helicobacter pylori exposure promotes development of CD4+ CD25+ FoxP3+ regulatory T cells. Infect Immun 75: 4334–4341. doi: 10.1128/IAI.00553-07 17562772 10.1128/IAI.00553-07 PMC1951191

[30] Das S, Suarez G, Beswick EJ, Sierra JC, Graham DY, et al (2006) Expression of B7-H1 on gastric epithelial cells: Its potential role in regulating T cells during Helicobacter pylori infection. Journal of Immunology 176: 3000–3009. 10.4049/jimmunol.176.5.3000 16493058

[31] Buddhisa S, Rinchai D, Ato M, Bancroft GJ, Lertmemongkolchai G (2015) Programmed death ligand 1 on Burkholderia pseudomallei-infected human polymorphonuclear neutrophils impairs T cell functions. J Immunol 194: 4413–4421. doi: 10.4049/jimmunol.1402417 25801435 10.4049/jimmunol.1402417

[32] Thibult ML, Mamessier E, Gertner-Dardenne J, Pastor S, Just-Landi S, et al (2013) PD-1 is a novel regulator of human B-cell activation. International Immunology 25: 129-+. doi: 10.1093/intimm/dxs098 23087177 10.1093/intimm/dxs098

[33] Benson DM, Bakan CE, Mishra A, Hofmeister CC, Efebera Y, et al (2010) The PD-1/PD-L1 axis modulates the natural killer cell versus multiple myeloma effect: a therapeutic target for CT-011, a novel monoclonal anti-PD-1 antibody. Blood 116: 2286–2294. doi: 10.1182/blood-2010-02-271874 20460501 10.1182/blood-2010-02-271874 PMC3490105

[34] Alvarez IB, Pasquinelli V, Jurado JO, Abbate E, Musella RM, et al (2010) Role Played by the Programmed Death-1-Programmed Death Ligand Pathway during Innate Immunity against Mycobacterium tuberculosis. Journal of Infectious Diseases 202: 524–532. doi: 10.1086/654932 20617899 10.1086/654932

[35] Okazaki T, Maeda A, Nishimura H, Kurosaki T, Honjo T (2001) PD-1 immunoreceptor inhibits B cell receptor-mediated signaling by recruiting src homology 2-domain-containing tyrosine phosphatase 2 to phosphotyrosine. Proc Natl Acad Sci U S A 98: 13866–13871. doi: 10.1073/pnas.231486598 11698646 10.1073/pnas.231486598 PMC61133

[36] Qorraj M, Bruns H, Bottcher M, Weigand L, Saul D, et al (2016) The PD-1/PD-L1 axis contributes to immune metabolic dysfunctions of monocytes in chronic lymphocytic leukemia. Leukemia. 10.1038/leu.2016.214 27479178

[37] Huang X, Venet F, Wang YL, Lepape A, Yuan Z, et al (2009) PD-1 expression by macrophages plays a pathologic role in altering microbial clearance and the innate inflammatory response to sepsis. Proc Natl Acad Sci U S A 106: 6303–6308. doi: 10.1073/pnas.0809422106 19332785 10.1073/pnas.0809422106 PMC2669369

[38] Larsson M, Shankar EM, Che KF, Saeidi A, Ellegard R, et al (2013) Molecular signatures of T-cell inhibition in HIV-1 infection. Retrovirology 10: 31 doi: 10.1186/1742-4690-10-31 23514593 10.1186/1742-4690-10-31 PMC3610157

[39] Sharpe AH, Freeman GJ (2002) The B7-CD28 superfamily. Nat Rev Immunol 2: 116–126. doi: 10.1038/nri727 11910893 10.1038/nri727

[40] Ye B, Liu X, Li X, Kong H, Tian L, et al (2015) T-cell exhaustion in chronic hepatitis B infection: current knowledge and clinical significance. Cell Death Dis 6: e1694 doi: 10.1038/cddis.2015.42 25789969 10.1038/cddis.2015.42 PMC4385920

[41] Anderson KM, Czinn SJ, Redline RW, Blanchard TG (2006) Induction of CTLA-4-mediated anergy contributes to persistent colonization in the murine model of gastric Helicobacter pylori infection. Journal of Immunology 176: 5306–5313. 10.4049/jimmunol.176.9.5306 16621997

[42] Quandt D, Hoff H, Rudolph M, Fillatreau S, Brunner-Weinzierl MC (2007) A new role of CTLA-4 on B cells in thymus-dependent immune responses in vivo. Journal of Immunology 179: 7316–7324. 10.4049/jimmunol.179.11.7316 18025174

[43] Pioli C, Gatta L, Ubaldi V, Doria G (2000) Inhibition of IgG1 and IgE production by stimulation of the B cell CTLA-4 receptor. J Immunol 165: 5530–5536. 11067906 10.4049/jimmunol.165.10.5530

[44] Laurent S, Carrega P, Saverino D, Piccioli P, Camoriano M, et al (2010) CTLA-4 is expressed by human monocyte-derived dendritic cells and regulates their functions. Human Immunology 71: 934–941. doi: 10.1016/j.humimm.2010.07.007 20650297 10.1016/j.humimm.2010.07.007

[45] Stojanovic A, Fiegler N, Brunner-Weinzierl M, Cerwenka A (2014) CTLA-4 Is Expressed by Activated Mouse NK Cells and Inhibits NK Cell IFN-gamma Production in Response to Mature Dendritic Cells. Journal of Immunology 192: 4184–4191. 10.4049/jimmunol.1302091 24688023

[46] See JX, Samudi C, Saeidi A, Menon N, Choh LC, et al (2016) Experimental Persistent Infection of BALB/c Mice with Small-Colony Variants of Burkholderia pseudomallei Leads to Concurrent Upregulation of PD-1 on T Cells and Skewed Th1 and Th17 Responses. PLoS Negl Trop Dis 10: e0004503 doi: 10.1371/journal.pntd.0004503 26974441 10.1371/journal.pntd.0004503 PMC4790896

[47] Ramli NS, Eng Guan C, Nathan S, Vadivelu J (2012) The effect of environmental conditions on biofilm formation of Burkholderia pseudomallei clinical isolates. PLoS One 7: e44104 doi: 10.1371/journal.pone.0044104 22970167 10.1371/journal.pone.0044104 PMC3435415

[48] Suppiah J, Thimma JS, Cheah SH, Vadivelu J (2010) Development and evaluation of polymerase chain reaction assay to detect Burkholderia genus and to differentiate the species in clinical specimens. FEMS Microbiol Lett 306: 9–14. doi: 10.1111/j.1574-6968.2010.01923.x 20345378 10.1111/j.1574-6968.2010.01923.x

[49] Goodyear A, Bielefeldt-Ohmann H, Schweizer H, Dow S (2012) Persistent gastric colonization with Burkholderia pseudomallei and dissemination from the gastrointestinal tract following mucosal inoculation of mice. PLoS One 7: e37324 doi: 10.1371/journal.pone.0037324 22624016 10.1371/journal.pone.0037324 PMC3356274

[50] Shankar EM, Che KF, Messmer D, Lifson JD, Larsson M (2011) Expression of a broad array of negative costimulatory molecules and Blimp-1 in T cells following priming by HIV-1 pulsed dendritic cells. Mol Med 17: 229–240. doi: 10.2119/molmed.2010.00175 21103670 10.2119/molmed.2010.00175 PMC3060986

[51] Che KF, Shankar EM, Muthu S, Zandi S, Sigvardsson M, et al (2012) p38 Mitogen-activated protein kinase/signal transducer and activator of transcription-3 pathway signaling regulates expression of inhibitory molecules in T cells activated by HIV-1-exposed dendritic cells. Mol Med 18: 1169–1182. doi: 10.2119/molmed.2012.00103 22777388 10.2119/molmed.2012.00103 PMC3510300

[52] Ahmad F, Hong HS, Jackel M, Jablonka A, Lu IN, et al (2014) High frequencies of polyfunctional CD8+ NK cells in chronic HIV-1 infection are associated with slower disease progression. J Virol 88: 12397–12408. doi: 10.1128/JVI.01420-14 25122796 10.1128/JVI.01420-14 PMC4248911

[53] Filion LG, Izaguirre CA, Garber GE, Huebsh L, Aye MT (1990) Detection of surface and cytoplasmic CD4 on blood monocytes from normal and HIV-1 infected individuals. J Immunol Methods 135: 59–69. 1703191 10.1016/0022-1759(90)90256-u

[54] Ashdown LR (1979) An improved screening technique for isolation of Pseudomonas pseudomallei from clinical specimens. Pathology 11: 293–297. 460953 10.3109/00313027909061954

[55] Al-Maleki AR, Mariappan V, Vellasamy KM, Tay ST, Vadivelu J (2015) Altered Proteome of Burkholderia pseudomallei Colony Variants Induced by Exposure to Human Lung Epithelial Cells. PLoS One 10: e0127398 doi: 10.1371/journal.pone.0127398 25996927 10.1371/journal.pone.0127398 PMC4440636

[56] Al-Maleki AR, Mariappan V, Vellasamy KM, Shankar EM, Tay ST, et al (2014) Enhanced intracellular survival and epithelial cell adherence abilities of Burkholderia pseudomallei morphotypes are dependent on differential expression of virulence-associated proteins during mid-logarithmic growth phase. J Proteomics 106: 205–220. doi: 10.1016/j.jprot.2014.04.005 24742602 10.1016/j.jprot.2014.04.005

[57] Tuchscherr L, Heitmann V, Hussain M, Viemann D, Roth J, et al (2010) Staphylococcus aureus small-colony variants are adapted phenotypes for intracellular persistence. J Infect Dis 202: 1031–1040. doi: 10.1086/656047 20715929 10.1086/656047

[58] Conejero L, Patel N, de Reynal M, Oberdorf S, Prior J, et al (2011) Low-dose exposure of C57BL/6 mice to burkholderia pseudomallei mimics chronic human melioidosis. Am J Pathol 179: 270–280. doi: 10.1016/j.ajpath.2011.03.031 21703409 10.1016/j.ajpath.2011.03.031 PMC3123849

[59] Currie BJ, Ward L, Cheng AC (2010) The epidemiology and clinical spectrum of melioidosis: 540 cases from the 20 year Darwin prospective study. PLoS Negl Trop Dis 4: e900 doi: 10.1371/journal.pntd.0000900 21152057 10.1371/journal.pntd.0000900 PMC2994918

[60] Dietrich J, Roy S, Rosenkrands I, Lindenstrom T, Filskov J, et al (2015) Differential influence of nutrient-starved Mycobacterium tuberculosis on adaptive immunity results in progressive tuberculosis disease and pathology. Infect Immun 83: 4731–4739. doi: 10.1128/IAI.01055-15 26416911 10.1128/IAI.01055-15 PMC4645392

[61] Cheng AC, Wuthiekanun V, Limmathurotsakul D, Chierakul W, Peacock SJ (2008) Intensity of exposure and incidence of melioidosis in Thai children. Trans R Soc Trop Med Hyg 102 Suppl 1: S37–39. 19121683 10.1016/S0035-9203(08)70010-5

[62] Macpherson AJ, Lamarre A, McCoy K, Harriman GR, Odermatt B, et al (2001) IgA production without mu or delta chain expression in developing B cells. Nat Immunol 2: 625–631. doi: 10.1038/89775 11429547 10.1038/89775

[63] Ghosh S, Hoselton SA, Schuh JM (2012) mu-chain-deficient mice possess B-1 cells and produce IgG and IgE, but not IgA, following systemic sensitization and inhalational challenge in a fungal asthma model. J Immunol 189: 1322–1329. doi: 10.4049/jimmunol.1200138 22732592 10.4049/jimmunol.1200138 PMC3401271

[64] Perona-Wright G, Mohrs K, Taylor J, Zaph C, Artis D, et al (2008) Cutting edge: Helminth infection induces IgE in the absence of mu- or delta-chain expression. J Immunol 181: 6697–6701. 18981085 10.4049/jimmunol.181.10.6697 PMC3066072

[65] Melamed D, Miri E, Leider N, Nemazee D (2000) Unexpected autoantibody production in membrane Ig-mu-deficient/lpr mice. J Immunol 165: 4353–4358. 11035071 10.4049/jimmunol.165.8.4353

[66] Haque A, Easton A, Smith D, O'Garra A, Van Rooijen N, et al (2006) Role of T cells in innate and adaptive immunity against murine Burkholderia pseudomallei infection. J Infect Dis 193: 370–379. doi: 10.1086/498983 16388484 10.1086/498983

[67] Barnes JL, Ketheesan N (2007) Development of protective immunity in a murine model of melioidosis is influenced by the source of Burkholderia pseudomallei antigens. Immunol Cell Biol 85: 551–557. doi: 10.1038/sj.icb.7100084 17563759 10.1038/sj.icb.7100084

[68] Sun JC, Lopez-Verges S, Kim CC, DeRisi JL, Lanier LL (2011) NK cells and immune "memory". J Immunol 186: 1891–1897. doi: 10.4049/jimmunol.1003035 21289313 10.4049/jimmunol.1003035 PMC4410097

[69] Cerwenka A, Lanier LL (2016) Natural killer cell memory in infection, inflammation and cancer. Nat Rev Immunol 16: 112–123. doi: 10.1038/nri.2015.9 26806484 10.1038/nri.2015.9

[70] da Silva IP, Gallois A, Jimenez-Baranda S, Khan S, Anderson AC, et al (2014) Reversal of NK-cell exhaustion in advanced melanoma by Tim-3 blockade. Cancer Immunol Res 2: 410–422. doi: 10.1158/2326-6066.CIR-13-0171 24795354 10.1158/2326-6066.CIR-13-0171 PMC4046278

[71] Gill S, Vasey AE, De Souza A, Baker J, Smith AT, et al (2012) Rapid development of exhaustion and down-regulation of eomesodermin limit the antitumor activity of adoptively transferred murine natural killer cells. Blood 119: 5758–5768. doi: 10.1182/blood-2012-03-415364 22544698 10.1182/blood-2012-03-415364 PMC3382935

[72] Jenjaroen K, Chumseng S, Sumonwiriya M, Ariyaprasert P, Chantratita N, et al (2015) T-Cell Responses Are Associated with Survival in Acute Melioidosis Patients. PLoS Negl Trop Dis 9: e0004152 doi: 10.1371/journal.pntd.0004152 26495852 10.1371/journal.pntd.0004152 PMC4619742

[73] Morris J, Williams N, Rush C, Govan B, Sangla K, et al (2012) Burkholderia pseudomallei triggers altered inflammatory profiles in a whole-blood model of type 2 diabetes-melioidosis comorbidity. Infect Immun 80: 2089–2099. doi: 10.1128/IAI.00212-12 22473609 10.1128/IAI.00212-12 PMC3370601

[74] Wiersinga WJ, Dessing MC, Kager PA, Cheng AC, Limmathurotsakul D, et al (2007) High-throughput mRNA profiling characterizes the expression of inflammatory molecules in sepsis caused by Burkholderia pseudomallei. Infect Immun 75: 3074–3079. doi: 10.1128/IAI.01733-06 17371859 10.1128/IAI.01733-06 PMC1932877

[75] Kulsantiwong P, Pudla M, Boondit J, Wikraiphat C, Dunachie SJ, et al (2016) Burkholderia pseudomallei induces IL-23 production in primary human monocytes. Med Microbiol Immunol 205: 255–260. doi: 10.1007/s00430-015-0440-z 26563410 10.1007/s00430-015-0440-z

[76] Korn T, Bettelli E, Oukka M, Kuchroo VK (2009) IL-17 and Th17 Cells. Annu Rev Immunol 27: 485–517. doi: 10.1146/annurev.immunol.021908.132710 19132915 10.1146/annurev.immunol.021908.132710

